# The provider’s checklist to improve pregnant women coverage by intermittent preventive malaria treatment in Mali: a pilot implementation study

**DOI:** 10.1186/s12936-021-03940-7

**Published:** 2021-10-16

**Authors:** Issa Doumbia, Fomba Seydou, Koné Diakalia, Issam Bennis

**Affiliations:** 1 Human Resources Directorate, Health and Social Development Ministry, Bamako, Mali; 2 National Malaria Control Program Directorate, Health and Social Development Ministry, Bamako, Mali; 3 National Control Program Malaria Directorate, Health and Social Development Ministry, Bamako, Mali; 4 Regional Directorate of the Ministry of Health and Social Protection, Fez, Morocco

**Keywords:** Malaria, Pregnant women, Implementation strategy, Intermittent preventive treatment, Sulfadoxine–pyrimethamine, Checklist, Mali

## Abstract

**Background:**

Intermittent preventive treatment of malaria in pregnancy (IPTp) is a comprehensive treatment protocol of anti-malarial drugs administered to pregnant women to prevent malaria, started at the fourth pregnancy month, with at least three doses of sulfadoxine–pyrimethamine (SP), taken as directly observed treatment (DOT) every 30 days at intervals until childbirth, in combination with other preventive measures. This paper introduces feasibility and adoption concepts as implementation research outcomes (IRO), allowing after a defined intervention, to assess the coverage improvement by IPTp for women attending a reference district hospital in Mali. Specifically, the purpose is to evaluate the feasibility of a reminder tool (provider checklist) to enhance pregnant women’s adoption of information about IPTp-SP uptake as immediate and sustained women practices.

**Methods:**

The implementation strategy used a reminder checklist about malaria knowledge and the recommended preventive tools. Then, the checklist feasibility was assessed during routine practices with the adoption-level about pregnant women’ knowledge. Quantitative data were collected through a questionnaire distributed to a non-probability purposive sampling targeting 200 pregnant women divided into two groups before and after the checklist intervention. In contrast, the qualitative data were based on in-depth face-to-face gynaecologists’ interviews.

**Results:**

Both the IROs (feasibility and adoption) were satisfactory. The gynaecologists agreed to the use of this checklist during routine practice with a recommendation to generalize it to other health providers. After a gynaecologist visit, a significant increase of the adoption-level about prior knowledge and preventive tools was noticed. A total of 83% of participants were not knowledgeable about malaria disease before checklist use *versus* 15% after. Similarly, coverage of women’s SP DOT rose from 0 to 59% after introducing the checklist and the IPTp-SP uptake after the visit was highly significant in the second group. The latter reached 95% of pregnant women with 4–8 months’ gestational age, that mostly respected all SP future visits as theoretically scheduled.

**Conclusions:**

Generalizing such a checklist reminder will improve women’s knowledge about malaria prevention.

**Supplementary Information:**

The online version contains supplementary material available at 10.1186/s12936-021-03940-7.

## Background

Malaria is a significant public health problem affecting more than 91 countries worldwide. According to the 2019 World Malaria Report, 85% of the global burden of malaria occurs in 19 sub-Saharan African countries and India. The incidence rate declined globally between 2010 and 2018 [1]. However, malaria cases were estimated at 228 million in 2018, with 405,000 associated deaths [1].

Pregnant women and children under 5 years old are the most affected [2, 3]. In sub-Saharan Africa, it is estimated that 25 to 30 million women are at risk of contracting *Plasmodium falciparum* during pregnancy [4]. Among the multiple prevention strategies identified, intermittent preventive treatment (IPTp) with sulfadoxine–pyrimethamine (SP) is recommended by the World Health Organization (WHO) in *P. falciparum-*stable transmission areas as an effective intervention [5]. This IPTp consists of administering at least three doses of SP in pregnancy from the 4th month until delivery, with at least one month between the different doses. The SP first dose is administered to pregnant women under directly observed treatment (DOT) [6]. This intervention’s effectiveness as preventive treatment is provided to reduce maternal malaria episodes, maternal and foetal anaemia, placental parasitaemia, low birth weight, and neonatal mortality [7, 8]. Due to this intervention, the prevalence of malaria parasitaemia in northeast Nigeria has been reduced by 40%, anaemia by 41%, and low birth weight by 37% [9].

Despite improved access to anti-malarial interventions, only 31% of pregnant women in 20 eligible countries had received at least three SP doses during their pregnancy in 2015 [10]. Moreover, it is already known that pregnant women do not receive relevant information about the appropriate timing to take preventive malaria drugs [11].

Based on the latest malaria report in Mali published in 2016, malaria affects the whole country and constitutes 32% of reasons for prior medical consultation. Health facilities and community health worker (CHW) sites have recorded more than two million confirmed cases, with a quarter of them severe cases. Pregnant women and children under 5 years old are the most affected by this disease [12]. In Mali and since 2003, IPTp with SP strategy has been implemented in both public and private sectors to reduce the consequences of malaria during pregnancy [13]. In 2006, the Ministry of Health introduced free IPTp with SP for pregnant women [14]. There has been progress between 2013 and 2018 with an increase from 15 to 55% coverage of pregnant women receiving three doses of SP during their last pregnancy. However, the overall targeted coverage (80%) is yet to be achieved. The lowest coverage (10%) was observed in Bamako District [13]. The main obstacle of using IPTp-SP highlighted the essential role of health professionals in promoting this coverage [13].

There is a need for a realistic implementation strategy to enhance the IPTp-SP coverage within this vulnerable category. Some successful implementation strategies to increase knowledge level are: firstly, increasing women’s attendance to antenatal consultations (ANC) since most women (52%) who did not receive IPTp-SP were those who did not attend ANC [15]. ANC is the official way to get free IPTp-SP. Secondly, using a provider checklist as a reminder information tool during ANC.

Many effective interventions in some contexts are not successful in other contexts due to ineffective implementation. Implementation research studies should clearly explain the targeted implementation outcomes (e.g., acceptability, adoption, appropriateness, feasibility, fidelity, implementation cost, penetration, sustainability) to measure the impact of the intervention used [16]. Subsequently, the study hypothesis is that the reminder checklist, as the intervention, will have feasible and adoptive outcomes in health facilities where a satisfactory ANC recruitment rate is achieved. The purpose of this implementation study is to assess routine practice feasibility of a reminder tool (provider checklist) in enhancing the adoption of information about IPTp-SP by pregnant women attending a reference district hospital with a high recruitment rate of ANC.

## Methods

### Design

**Fig. 1:**
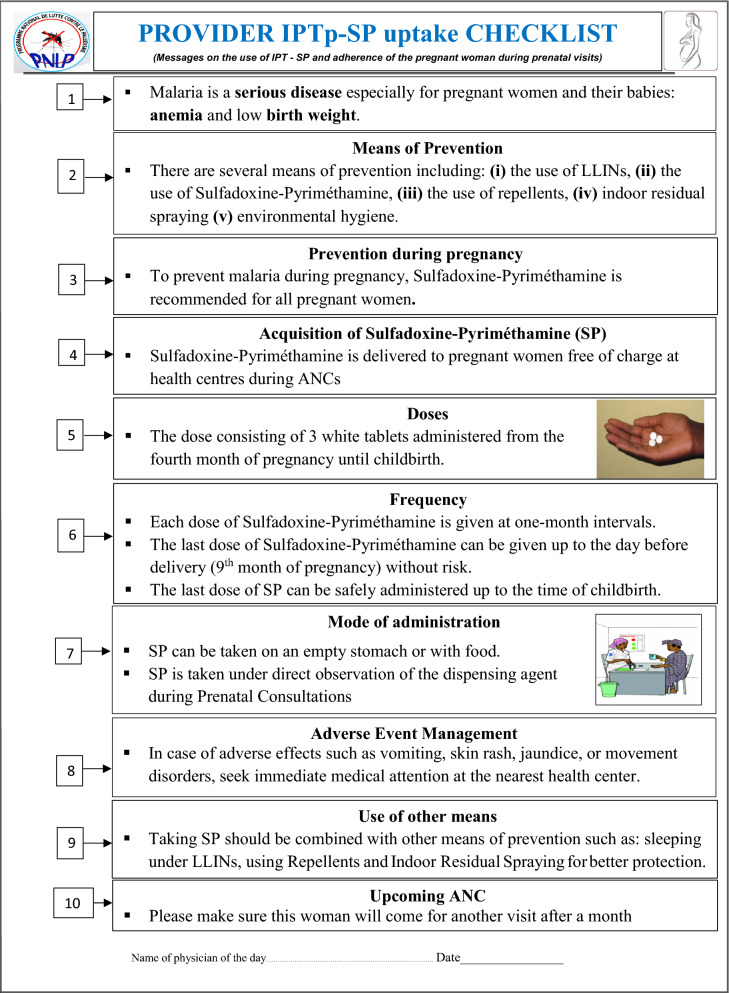
Provider IPTp-SP uptake checklist

This is an implementation study using an explanatory mixed-method as the first phase, quantitative data collection (QUAN), followed by a small qualitative data collection (qual), to explain the initial quantitative results. First, an implementation strategy based on a reminder checklist (Fig. 1) about malaria knowledge and recommended preventive tools was conducted. Then, the research team assessed the feasibility of such a checklist in routine practices and the adoption of information given by healthcare providers to pregnant women as immediate and sustained practices. The standards for reporting implementation studies: StaRI Checklist were followed (Additional file 2) [17].

### Outcome concepts

Feasibility is defined as the extent to which the checklist can be successfully used as a self-reminder tool which a health provider checks in front of a pregnant woman to give her 10 mandatory information points linked to malaria during pregnancy. Once the information was explained, a corresponding box was ticked by the gynaecologist (Fig. 1).

Adoption is defined as the eligible pregnant woman understanding the given preventive message of malaria risks and prevention, as her intention to take SP doses immediately in the drug unit before leaving the health facility, and as scheduled to return every month for the same purpose until childbirth. This adoption outcome could be measured from the perspective of the provider who confirms the usefulness of DOT for eligible pregnant women or, retrospectively, from the perspective of the organization by seeking the SP notebook (ID, actual and future dates of visit, pregnancy age, dates of IPTp-SP uptakes) and the sustained SP visits until childbirth by phone call (Additional file 3).

### Context and study site

The study was conducted at the District Hospital of Commune VI in Bamako, located beside the River Niger, and at 94 sq km, the largest commune in the District of Bamako. The hospital recorded a high recruitment rate in ANC, enrolling 130,675 pregnant women’s visits during 2016–2017; in a population estimated at 611,392 inhabitants and 30,570 pregnant women.

### Target population

The study was conducted between February and April 2018. The inclusion criteria were women between 15 and 49 years old; with a pregnancy of four months or more determined by the result of obstetric ultrasound; living in the same commune of the district hospital; and, who came to the health facility for programmed antenatal visits. The exclusion criteria were the non-eligibility criteria that affirmed an SP allergy or side effects.

Non-probability purposive sampling was applied. All pregnant women with study eligibility criteria were recruited during the study by targeting 100 pregnant women before and another 100 after the checklist intervention. Four gynaecologists (A, B, C, D) who were ‘blinded’ to the existence of the checklist were invited to address all eligible pregnant women at the end of their health facility visit at the drug unit where the women received appropriate medication. If SP was recommended, DOT SP should be taken in front of a community health worker or a nurse at the unit. Then, pregnant women who wanted to participate in the study met the study investigator. In another room respecting confidentiality, each pregnant woman who had already received the drug prescription was officially invited to participate in the study and provided a written consent approval before starting face-to-face interview with the study investigator (first author). All gave phone numbers for follow-up calls and received advice to follow the pathway recommended by the health professionals without any intervention in the process. A full description of the implementation process is included as Additional file 5.

Once the first 100 women were enrolled, the investigator invited the same gynaecologists to introduce the checklist provider during the ANC and continue sending eligible pregnant women on the same pathway. The second group consisted of an additional 100 women who received the provider checklist messages during the antenatal gynaecologist consultation. To increase data reliability in this study, the proportions of pregnant women recruited by each gynaecologist were identical in both phases. The 200 women selected were determined based on budgetary and time constraints, considering the days of consultations of each gynaecologist participating in the District Hospital of Commune VI during the study’s period.

For the qualitative section, the in-depth interviews with the gynaecologists were to explain the remaining questions arising from the analysis of the quantitative section.

### Data collection plan

Three tools were used to collect information before and after the introduction of the implementation strategy intervention (consisting of a provider checklist to remind the gynaecologists to share key messages about IPTp-SP uptake with pregnant women during the ANC visit):

- 1st tool: A quantitative questionnaire was administered to all participating pregnant women before leaving the health facility to assess the newest information provided by physicians at ANCs and assess the women’s malaria knowledge (Additional file 4).
- 2nd tool: A qualitative, thematic, in-depth interview guide with all participating gynaecologists at the end of data collection. The thematic guide targeted eight questions about the checklist provider’s usefulness and feasibility: (1) are there any comments about its content?; (2) how can it be improved?; (3) did it allow you to remember the information transmitted to the pregnant woman during the ANC?; (4) in your opinion, does the checklist help improve the knowledge of pregnant women in the context of IPTp-SP?; (5) if such a checklist is widespread in all reference health centres, what do you think would be the obstacles to use it?; (6) was the transmission of the checklist messages impacting your time?; (7) in general, do you think the checklist is a good tool to support women’s adherence and adoption to the supervised SP?; and, (8) who would be the best category(ies) of health professionals for its use (gynaecologist, general practitioner, midwife, nurse in charge of pharmacy, another profile to specify, please)?
- 3rd tool: An observational sheet was used to document participants’ IPTp-SP uptake in front of a health provider or a community health worker inside the health facility. The investigator checked the SP information on the drug unit’s dispensing record at the end of each study day. One year later, the investigator telephoned all participating women to check their pregnancy outcome, the effective date and whether they took SP in the months before childbirth, how many times and so on. All this information is available in Additional file 3.

### Data analysis

The quantitative data were entered and analysed in the IBM statistical SPSS software version 20.0. Pregnant women’s knowledge of malaria was analysed by classifying them into three groups, combining their knowledge of malaria symptoms and means of prevention. The current IPTp-SP uptake by pregnant women under direct observation was assessed before and after introducing the checklist. Sociodemographic characteristics were also grouped into categories. Accurate Chi-square/Fisher tests were performed for the use of the checklist compared to the study variables. Any association with a p-value < 0.05 was considered significant.

The quantitative improvement of knowledge about malaria in participating women and the qualitative information about the ease of use of this checklist by gynaecologists in their routine ANC practices were targeted to assess the provider checklist’s feasibility.

Similarly, to assess the adoption by pregnant women of IPTp-SP information, the quantitative ratio of pregnant women who decided to take DOT at the drug unit before leaving the health facility, and the qualitative explanation for any misunderstanding of the results, such as the number of pregnant women not well informed even after the use of the checklist, were analysed. The main information about adoption was calculated by comparing the due overall IPTp-SP uptake times and real times of IPTp-SP uptake noted prospectively by phone calls.

The classification of women’s malaria knowledge adoption level was considered:

- Very good: For a woman who responded with certainty that fever is the main malaria symptom and knew the four protective WHO measures against malaria (long-lasting impregnated mosquito net; indoor residual spraying; indoor repellent; and IPTp-SP uptake).
- Good: For a woman who cited fever as the main malaria symptom and knew at least two protective WHO measures.
- Average: For a woman who cited fever as the main malaria symptom and knew only one protective WHO measure.
- Did not know: For a woman who cited fever as the main malaria symptom but did not know of protective WHO measures; or for a woman who did not know fever is the main malaria symptom nor the protective WHO measures.

The qualitative, in-depth, audio-recorded interviews with the gynaecologists allowed an understanding of the overall pregnant women pathway. The translated transcripts of the interviews were analysed by NVivo version 11 international QRS software, and a resumé of thematic analysis was developed following Bazely recommendations [18]. The last author of this paper (IB) defined the main themes firstly after reading the transcript; then, the first author (ID) refined the data by linking quotes to the themes. After that, IB and ID discussed the tree quotes meaning similarities and differences to make the final coding decision.

## Results

### Quantitative results

Two-hundred participants meeting the inclusion criteria were included in the study. The duration of the questionnaire administration to each participant was approximately 10 ± 0 min. Nine pregnant women declined to participate due to family duties and were not included in the analysis (Additional file 1). All were between 15 and 42 years old. The average number of pregnancies was four, with a maximum of 12. The average number of ANC visits among participating women was four, with a maximum of eight. The average concentration of sample haemoglobin level was 11.3 ± 1.3 g/dl, with a maximum of 14.7 g/dl. Out of 200 pregnant women, 11 and 15 were anaemic in both groups. Other sociodemographic of the participating women are presented in Table 1. None of the characteristics was statistically significant in before and after checklist comparison.

**Table 1:** Sociodemographic and clinical characteristics of participating pregnant women

Characteristics	Before the checklist (n = 100)	After the checklist (n = 100)	*p*-value
Women age			
15–25 years	48	41	0.32
≥ 26 years	52	59	
Marital status			
Married	99	97	0.62
Single	1	3	
Literacy			
Illiterate	30	26	0.53
Literate	70	74	
Cultural environment			
Bambara	37	40	0.66
Peuhl & others	63	60	
Gestation number			
1 to 3 gestations	61	67	0.38
Four and over	39	33	
Gestational age			
4 to 8 months	75	69	0.34
≥ 9 months	25	31	

The pregnant women in the first group had the right to visit any gynaecologist depending on the daily schedule availability. The final repartition among the four gynaecologists was: A (35), B (30), C (20), and D (15). The same ratio of allocation was respected in the second group. The study’s main result shows that the checklist impacts malaria knowledge independently of the gynaecologists. Indeed, there is a statistically significant relationship between the physicians’ checklist use and pregnant women’s correct responses about malaria information (Table 2).

**Table 2:** Distribution of pregnant women per gynaecologist

Physician	Women number	Number of pregnant women knowledgeable about malaria				*p*-value
		Before the checklist		After the checklist		
		Yes	No	Yes	No	
A	35 × 2	5	30	29	6	< 0.001
B	30 × 2	4	26	29	1	< 0.001
C	20 × 2	4	16	13	7	< 0.01
D	15 × 2	4	11	14	1	< 0.001
Total	200	17	83	85	15	< 0.001

The women’s knowledge was assessed according to the type of combination of malaria symptoms and preventions, and the information given during the checklist reminder was statistically significant (Table 3). When the checklist was used, good and very good knowledge improved between the two groups from 2 to 39%. Compared to the knowledge of conditions of compliance needed to take three SP doses, the correct response rate increased in the group of women benefiting from the checklist intervention. In addition, the relationship between supervised IPTp-SP uptake and pregnant women was statistically significant between the two groups. According to Table 4, the coverage rate of supervised IPTp-SP uptake increased from 0 to 59%.

**Table 3:** Distribution of women in terms of knowledge of malaria disease before and after the checklist

Symptom and means of prevention	Knowledge		
	Before the checklist n = 100	After the checklist n = 100	*p* value
Average (Fever + LLIN)	21	0	
Good (Fever + LLIN + IPTp-SP)	2	17	0.01*
Very good (Fever + LLIN + IPTp-SP + Rep. /IRS)	0	22	
Does not know (Fever + no preventive means)	77	61	

*LLIN* Long lasting impregnated mosquito net, *IRS* Indoor residual spraying, *Rep* Repellent

*p-value was calculated by fisher test comparing the association of average, good and very good replies number, versus do not know the number

**Table 4:** Distribution of the immediate adoption by pregnant women of supervised IPTp-SP uptake of the three doses during their facility visit

Number of women who adopt the supervised use of the 3 SP doses	Before the checklist n = 100	After the checklist n = 100	*p*-value
Yes	0	59	< 0.001
No	100	41	

Reasons for not taking SP during pregnant women according to their degree of information	n = 100	n = 41/100	
I have not eaten yet, and I was told that I could take it at home after a meal to minimize side effects	75	27	
The doctor did not explain to me that I must take it immediately here	20	5	
There is no water at the facility to take the treatment	0	1	
I prefer to take it at home (with more hygiene)	5	7	
I must take a blood sample before taking the treatment	0	1	

In the qualitative analysis, the drug unit’s community health worker was unfortunately identified as the source of contradictory information, advising women to take the SP after a meal to avoid side effects, in contradiction to WHO recommendations and Mali’s national guidelines IPTp-SP. Some 75% of women from the first group *versus* 41% from the second group did not take SP for this reason. Luckily, the other IPTp-SP uptake visits were confirmed as completed by phone calls in more than 95% in the second group compared to only 38% in the first group. Table 5 informs about the effective IPTp-SP uptake compared to the scheduled SP-post-study uptake until childbirth days of those pregnant women recruited with a gestational age between four and eight months.

**Table 5:** The effective IPTp-SP uptake compared to the scheduled IPTp-SP post-study uptakes until childbirth days of the pregnant women recruited with a gestational age between four and eight months

Women who confirmed by phone the total SP intakes	Before the checklist n = 50^a^	After the checklist n = 69	*P*-Value
Correct number like the theoretical due IPTp-SP uptake date	19	66	< 0.0001
Not a correct number, fewer than the theoretical due IPTp-SP uptake date	31	03	

We excluded in this table the 9 months and above pregnant women because they had to take almost zero next SP (NB: Even by adding all participants the difference remains highly significant)

^a^25 women do not reply to the phone call or changed the phone number

During the study, all women (200) were asked about the benefit of providers’ communication at ANC to understand health messages. Almost 90% considered that good communication in general is essential, and 162 women confirmed the importance of allocating the necessary time by physicians during ANC visits to discuss malaria prevention and pregnancy, defining the needed adequate time between 10 and 20 min. Those responses were similar for women in both phases of the study.

### Qualitative results

In the qualitative part of the study, participating gynaecologists agreed that the checklist helped them remember all messages transmitted to pregnant woman during ANC visits. They confirmed that they allowed more or less time to explain malaria risks based on previous women knowledge and life experiences. This qualitative part helped in understanding the quantitative results presented in Table 3, noting that 77 women in the non-checklist group were not aware of malaria preventive tools compared to 61 women in the checklist group. In the first group, all pregnant women took SP during their previous visits without knowing the relation between the drug and malaria prevention. It was considered a ‘vitamin’ pill to sustain a healthy pregnancy state. However, for 61 pregnant women from the group receiving checklist provider explanations and giving ‘do not know’ response about SP, it was mainly linked to SP’s presentation by the gynaecologists, as spoken information without seeing the SP pills boxes. While at the study-investigator meeting, the women were asked if during the questionnaire they knew SP by showing them three different existing commercial boxes of the drug. That was why one gynaecologist suggested adding full pictures of the commercial name of SP into the checklist provider future version. Consequently, if the study investigator asked them about SP showing the same pillboxes, they would respond to knowing one preventive measure; a statistically significant difference would appear.

Another gynaecologist suggested the introduction of the checklist by nurses and midwives during the unit ANC visits. However, the lack of full explanations about malaria prevention is due to the repetitive process of such messages *versus* workload conditions. Indeed, it is challenging for health professionals to provide the same educational statement to each new visiting pregnant woman. To manage such situations, another health professional should periodically notice the quality of the information shared.

The DOT was in part not respected as some pregnant women were asked to take SP at home after a meal. The motivation was based first on the health worker’s conviction that taking SP with a good meal decreases side effect probabilities and avoids unnecessary future work if that woman returns to the health facility. For an IPTp-SP uptake-friendly atmosphere, there is a need to rethink the unit drug’s location, where an open and appropriate seating area is available with permanent access to potable water or clean water bottles, in order to help health workers, supervise women’s IPTp-SP uptake, independently of age or sociocultural backgrounds, without shyness or embarrassment.

## Discussion

In this study, the lack of knowledge about malaria pregnancy risks limited SP’s DOT. This result is consistent with another study conducted in India, in which 80% of pregnant women did not have information about malaria at the time of contact with health professionals [19]. The same observation was made in Benin, where half of pregnant women had not had how to take SP explained [20].

The use of a reminder provider checklist improved statistically, IPTp-SP-supervised uptake, by encouraging the provider to give malaria-structured information to visiting pregnant women. The simple, one-page checklist reduced women’s information gap in one contact during ANC. Moreover, due to a high level of trust in the doctor’s advice it was more appreciated and less unforgettable. In this study, the second group was the most respectful of all future SP visits and preventive measures, as theoretically scheduled for 95% of all pregnant women recruited with a gestational age between four and eight months. The adoption of IPTp-SP uptake by pregnant women was highly significant compared with the first group.

The feasibility and adoption of this intervention were proved in the context of this study. A recent qualitative study from Mozambique highlighted the need to foster health education and information sources against malaria risks in pregnancy for both health professionals and pregnant women [21].

The participating women with the checklist, whatever their level of previous knowledge or education, understood that SP is a preventive and non-curative drug designed to protect them during pregnancy and protect their new-borns, as a study from India found out [19]. The checklist’s usefulness is consistent with a study in Nigeria which found that both women’s knowledge and education improvement impacted malaria control [22].

Such results confirm that for health facilities in Mali where ANC recruitment is highly achievable, fostering information about pregnancy risks and its prevention tools could help reach better coverage of IPTp-SP. For instance, the checklist reminder improved the immediate scope of more than half of the IPTp participants in real-life conditions, contextualizing this study by moving from 0 to 59% after its use. Simultaneously, women with 4 to 8 months of gestational age were the most respectful of future SP visits as theoretically scheduled in the second group (95%) compared to the first group (38%). The mean rate (59%) of pregnant women who enrolled in IPTp-SP is slightly higher than the results of a study from Burkina Faso (55%) [23]. This coverage rate is much higher than women who received at least three or more doses in a multicentric study done in 36 African countries where the improvement was slower at 31% in 2018, compared with 22% in 2017 versus 2% in 2010 [1].

Without supervision, community agents and health workers could confuse preventive treatment information targeting pregnant women [24, 25]. For instance, the contradictory message that suggested pregnant women take SP at home after a meal to minimize side effects was found in another study in Mali [14]. These communities should understand that malaria has harmful consequences for mother and her child and that administering DOT SP at specific times of pregnancy is one of the most effective ways, in addition to other preventive measures, to be protected [26]. The need for effective communication and understanding between health workers on the one hand and between health workers and their patients and communities on the other hand, are essential for increasing acceptability and adoption of the IPTp-SP [27]. A recent household study about the determinants of IPTp-SP in Mali confirmed the key role of communication in the early initiation of ANC, the accessibility to a community health centre, the ability to read, and knowledge of the utility of the drug [28].

Mali adopts WHO standards to define ANC coverage needs. Mali’s 2018–2022 National Malaria Control Strategic Plan targets the achievement of 80% of the use of the third dose of IPT-SP or higher. According to new WHO recommendations, contact between woman and provider must be more than just one ANC visit [29]. This provider checklist, as a new routine ANC tool associated with an extension by MHealth innovating technologies (reminder SMS, reminder calls), could systematically create more opportunities for giving complete information about malaria and pregnancy, helping achieve the desired results of ANC coverage. However, the under-reporting of IPTp-SP uptake by women who do not visit health facilities and take SP by themselves or take SP from other sources than those available at public health facilities mitigates the accuracy of coverage rate and needs more investigation [15].

This study has some limitations. Firstly, although the physicians confirmed giving the 10 points of information on the checklist, the study investigator did not have access to the full physician-woman discussion during ANC. Thus, it is impossible to verify if all ideas included in the checklist were explained in the same way and with the same time length. Secondly, the evaluation of the time spent by each physician for each woman was not assessed due to the variety of ANC motivations that included, in the same visits, other health questions than malaria prevention. Thirdly, the authors did not assess the effect of such a checklist on any increase in ANC visits and if it may improve the service cost-effectiveness. Finally, the study investigator was not blinded about the checklist use and had not planned to confirm SP’s source taken previously by some participants, if it was exclusively available in the health facility or had other sources.

## Conclusions

The ease of use of this checklist in daily practice increased women’s adoption of SP during facility visits in front of a health worker as recommended by the national programme. This provider checklist reminder tool can be updated and generalized as a pre-natal consultation activity in all public health facilities in Mali and similarly malaria-endemic countries. Based on this provider checklist’s encouraging results, further research is suggested to assess other outcomes: acceptability, coverage and sustainability at organizational level.

## Supplementary Information

**Additional file 1.** Study flow diagram.

**Additional file 2.** Study method StaRI checklist.

**Additional file 3.** Participating women characteristics with follow-up of the IPTp-SP uptake state.

**Additional file 4.** English version of full face-to-face, open-ended questionnaire for participating pregnant women.

**Additional file 5.** Study method additional information.

## Data Availability

All quantitative data analysed for this study are shared in open access within an Excel file available as additional material.

## Abbreviations

ANC: Antenatal care visit
ANC1: First antenatal care visit
ANC3: Third antenatal care visit
ANC4: Fourth antenatal care visit
ANC8: Eighth antenatal care visit
CHW: Community health worker
DOT: Directly observed treatment
IPTp-SP: Intermittent preventive treatment during pregnancy with sulfadoxine–pyrimethamine
IRO: Implementation research outcomes
IRS: Indoor residual spraying
LLIN: Long-lasting impregnated mosquito net
NMCP: National Malaria Control Programme
Rep: Repellent
SP: Sulfadoxine–pyrimethamine
Sq Km: Square kilometre
WHO: World Health Organization
