# Supplementary material for: The provider’s checklist to improve pregnant women coverage by intermittent preventive malaria treatment in Mali: a pilot implementation study
Source: Malar J. 2021 Oct 16;20:402. doi: 10.1186/s12936-021-03940-7 (PMC8520273; doi:10.1186/s12936-021-03940-7)
Supplement: Supplementary file 1 — Additional file 1. Study flow diagram. [file 12936_2021_3940_MOESM1_ESM.docx]

Enrollment

Assessed for eligibility (n= **984**)

Excluded (n= **784**)

♦ Not meeting inclusion criteria (n= 07)

♦ Declined to participate (n= 09 )

♦ Not meeting the gynecologist ANC availability days (n= 768) more information in the **supplementary material-5**

Recruited (n= **200**)

Allocation

Allocated to consultation without implementation of the checklist (n= 100)

♦ Received only consultation (n= 100 )

♦ Did not receive consultation only (n= 0 )

Allocated to consultation with implementation of the checklist (n= 100)

♦ Received consultation and checklist (n=100 )

♦ Did not receive consult +checklist (n=0 )

Follow-Up

Lost to IPTp-SP uptake in front of a health professional (n= **100**)

Reasons: See table III

Lost to IPTp-SP uptake in front of a health professional (n= **41**)

Reasons: See table III

**Analysed (n= 100)**

**Analysed (n= 100)**

Analysis

**Additional file-1** Study flow diagram
