# Supplementary material for: The provider’s checklist to improve pregnant women coverage by intermittent preventive malaria treatment in Mali: a pilot implementation study
Source: Malar J. 2021 Oct 16;20:402. doi: 10.1186/s12936-021-03940-7 (PMC8520273; doi:10.1186/s12936-021-03940-7)
Supplement: Supplementary file 3 — Additional file 3. Participating women characteristics with follow-up of the IPTp-SP uptake state. [file 12936_2021_3940_MOESM3_ESM.docx]

**Additional file-3** Participating women characteristics with follow-up of the IPTp-SP uptake state

| **The pregnant woman ID** | **Group*** | **Date of the visit** | **Range of previous gestures** | **Gestational age group** | **Actual Gestational age range** | **ANC number including the one performed during the study** | **SP DOT done correctly during the PI’s study visit contact** | **Final phone call to know if they took the SP regularly after the PI contact (**)** | **Theoretical remaining IPTp-SP uptake based on the ANC age before the childbirth** | **Effective SP times taken after the PI meeting** | **Comparison between the effective and theoretical IPTp-SP uptake** |
| --- | --- | --- | --- | --- | --- | --- | --- | --- | --- | --- | --- |
| 1 | 1 | 23/2 | 1 to 3 | ≥ 9 months | 9 months and more | 4 | No | **No** | 0 to 1 | 0 | Correct |
| 2 | 1 | 23/2 | Four and over | ≥ 9 months | 9 months and more | 4 | No | **No** | 0 to 1 | 0 | Correct |
| 3 | 1 | 23/2 | 1 to 3 | [4 to 8months] | Between 4 and 6 months | 1 | No | **Unknown** | 3 to 5 | No information | No |
| 4 | 1 | 23/2 | Four and over | ≥ 9 months | 9 months and more | 4 | No | **No** | 0 to 1 | 0 | Correct |
| 5 | 1 | 23/2 | Four and over | [4 to 8months] | Between 8 and 9 months | 5 | No | **Yes** | 1 to 2 | 1 | Correct |
| 6 | 1 | 23/2 | 1 to 3 | [4 to 8months] | Between 8 and 9 months | 6 | No | **Yes** | 1 to 2 | 1 | Correct |
| 7 | 1 | 26/2 | 1 to 3 | [4 to 8months] | Between 7 and 8 months | 4 | No | **Unknown** | 1 to 3 | No information | No |
| 8 | 1 | 26/2 | Four and over | [4 to 8months] | Between 8 and 9 months | 4 | No | **No** | 1 to 2 | 0 | No |
| 9 | 1 | 26/2 | 1 to 3 | [4 to 8months] | Between 8 and 9 months | 4 | No | **No** | 1 to 2 | 0 | No |
| 10 | 1 | 26/2 | 1 to 3 | [4 to 8months] | Between 7 and 8 months | 4 | No | **Yes** | 1 to 3 | 2 | Correct |
| 11 | 1 | 26/2 | 1 to 3 | [4 to 8months] | Between 4 and 6 months | 1 | No | **Yes** | 3 to 5 | 5 | Correct |
| 12 | 1 | 26/2 | 1 to 3 | [4 to 8months] | Between 8 and 9 months | 5 | No | **Yes** | 1 to 2 | 1 | Correct |
| 13 | 1 | 26/2 | 1 to 3 | [4 to 8months] | Between 4 and 6 months | 1 | No | **Unknown** | 3 to 5 | No information | Correct |
| 14 | 1 | 26/2 | 1 to 3 | ≥ 9 months | 9 months and more | 4 | No | **No** | 0 to 1 | 0 | Correct |
| 15 | 1 | 28/2 | 1 to 3 | ≥ 9 months | 9 months and more | 5 | No | **No** | 0 to 1 | 0 | Correct |
| 16 | 1 | 28/2 | 1 to 3 | ≥ 9 months | 9 months and more | 1 | No | **No** | 0 to 1 | 0 | Correct |
| 17 | 1 | 28/2 | 1 to 3 | [4 to 8months] | Between 7 and 8 months | 4 | No | **Unknown** | 1 to 3 | No information | No |
| 18 | 1 | 28/2 | Four and over | ≥ 9 months | 9 months and more | 4 | No | **No** | 0 to 1 | 0 | Correct |
| 19 | 1 | 28/2 | 1 to 3 | [4 to 8months] | Between 8 and 9 months | 3 | No | **No** | 1 to 2 | 0 | No |
| 20 | 1 | 28/2 | Four and over | ≥ 9 months | 9 months and more | 2 | No | **No** | 0 to 1 | 0 | Correct |
| 21 | 1 | 28/2 | Four and over | [4 to 8months] | Between 8 and 9 months | 2 | No | **No** | 1 to 2 | 0 | No |
| 22 | 1 | 28/2 | Four and over | [4 to 8months] | Between 7 and 8 months | 5 | No | **Yes** | 1 to 3 | 2 | Correct |
| 23 | 1 | 28/2 | Four and over | [4 to 8months] | Between 7 and 8 months | 3 | No | **Yes** | 1 to 3 | 2 | Correct |
| 24 | 1 | 28/2 | 1 to 3 | [4 to 8months] | Between 7 and 8 months | 3 | No | **Unknown** | 1 to 3 | No information | No |
| 25 | 1 | 28/2 | 1 to 3 | ≥ 9 months | 9 months and more | 4 | No | **No** | 0 to 1 | 0 | Correct |
| 26 | 1 | 1/3 | 1 to 3 | [4 to 8months] | Between 8 and 9 months | 2 | No | **Yes** | 1 to 2 | 1 | Correct |
| 27 | 1 | 1/3 | Four and over | [4 to 8months] | Between 8 and 9 months | 2 | No | **No** | 1 to 2 | 0 | No |
| 28 | 1 | 1/3 | Four and over | [4 to 8months] | Between 4 and 6 months | 3 | No | **Unknown** | 3 to 5 | No information | No |
| 29 | 1 | 5/3 | Four and over | [4 to 8months] | Between 7 and 8 months | 3 | No | **Yes** | 1 to 3 | 2 | Correct |
| 30 | 1 | 5/3 | 1 to 3 | [4 to 8months] | Between 7 and 8 months | 4 | No | **Unknown** | 1 to 3 | No information | No |
| 31 | 1 | 5/3 | Four and over | [4 to 8months] | Between 4 and 6 months | 1 | No | **Unknown** | 3 to 5 | No information | No |
| 32 | 1 | 5/3 | 1 to 3 | ≥ 9 months | 9 months and more | 5 | No | **No** | 0 to 1 | 0 | Correct |
| 33 | 1 | 5/3 | 1 to 3 | [4 to 8months] | Between 4 and 6 months | 2 | No | **Unknown** | 3 to 5 | No information | No |
| 34 | 1 | 5/3 | Four and over | [4 to 8months] | Between 8 and 9 months | 4 | No | **No** | 1 to 2 | 0 | No |
| 35 | 1 | 5/3 | 1 to 3 | [4 to 8months] | Between 8 and 9 months | 3 | No | **No** | 1 to 2 | 0 | No |
| 36 | 1 | 5/3 | 1 to 3 | [4 to 8months] | Between 8 and 9 months | 3 | No | **No** | 1 to 2 | 0 | No |
| 37 | 1 | 6/3 | 1 to 3 | [4 to 8months] | Between 7 and 8 months | 3 | No | **Yes** | 1 to 3 | 2 | Correct |
| 38 | 1 | 6/3 | Four and over | [4 to 8months] | Between 4 and 6 months | 3 | No | **Unknown** | 3 to 5 | No information | No |
| 39 | 1 | 6/3 | 1 to 3 | [4 to 8months] | Between 8 and 9 months | 2 | No | **Unknown** | 1 to 2 | No information | No |
| 40 | 1 | 6/3 | Four and over | ≥ 9 months | 9 months and more | 6 | No | **No** | 0 to 1 | 0 | Correct |
| 41 | 1 | 6/3 | Four and over | [4 to 8months] | Between 8 and 9 months | 3 | No | **No** | 1 to 2 | 0 | No |
| 42 | 1 | 6/3 | Four and over | ≥ 9 months | 9 months and more | 3 | No | **No** | 0 to 1 | 0 | Correct |
| 43 | 1 | 6/3 | 1 to 3 | [4 to 8months] | Between 8 and 9 months | 3 | No | **Yes** | 1 to 2 | 1 | Correct |
| 44 | 1 | 6/3 | Four and over | [4 to 8months] | Between 7 and 8 months | 4 | No | **Yes** | 1 to 3 | 2 | Correct |
| 45 | 1 | 6/3 | 1 to 3 | [4 to 8months] | Between 8 and 9 months | 4 | No | **No** | 1 to 2 | 0 | No |
| 46 | 1 | 6/3 | Four and over | [4 to 8months] | Between 8 and 9 months | 2 | No | **No** | 1 to 2 | 0 | No |
| 47 | 1 | 6/3 | 1 to 3 | [4 to 8months] | Between 7 and 8 months | 2 | No | **Unknown** | 1 to 3 | No information | No |
| 48 | 1 | 6/3 | 1 to 3 | [4 to 8months] | Between 4 and 6 months | 5 | No | **Yes** | 3 to 5 | 5 | Correct |
| 49 | 1 | 6/3 | Four and over | ≥ 9 months | 9 months and more | 2 | No | **No** | 0 to 1 | 0 | Correct |
| 50 | 1 | 6/3 | 1 to 3 | ≥ 9 months | 9 months and more | 5 | No | **No** | 0 to 1 | 0 | Correct |
| 51 | 1 | 6/3 | Four and over | [4 to 8months] | Between 8 and 9 months | 4 | No | **No** | 1 to 2 | 0 | No |
| 52 | 1 | 7/3 | Four and over | [4 to 8months] | Between 7 and 8 months | 5 | No | **Unknown** | 1 to 3 | No information | No |
| 53 | 1 | 7/3 | Four and over | ≥ 9 months | 9 months and more | 5 | No | **No** | 0 to 1 | 0 | Correct |
| 54 | 1 | 7/3 | 1 to 3 | [4 to 8months] | Between 8 and 9 months | 3 | No | **No** | 1 to 2 | 0 | No |
| 55 | 1 | 7/3 | 1 to 3 | ≥ 9 months | 9 months and more | 6 | No | **No** | 0 to 1 | 0 | Correct |
| 56 | 1 | 7/3 | 1 to 3 | [4 to 8months] | Between 8 and 9 months | 4 | No | **No** | 1 to 2 | 0 | No |
| 57 | 1 | 7/3 | Four and over | [4 to 8months] | Between 7 and 8 months | 2 | No | **Yes** | 1 to 3 | 2 | Correct |
| 58 | 1 | 7/3 | 1 to 3 | [4 to 8months] | Between 8 and 9 months | 5 | No | **No** | 1 to 2 | 0 | No |
| 59 | 1 | 7/3 | Four and over | [4 to 8months] | Between 7 and 8 months | 4 | No | **Unknown** | 1 to 3 | No information | No. |
| 60 | 1 | 7/3 | Four and over | [4 to 8months] | Between 8 and 9 months | 2 | No | **No** | 1 to 2 | 0 | No |
| 61 | 1 | 5/3 | Four and over | [4 to 8months] | Between 4 and 6 months | 1 | No | **Yes** | 3 to 5 | 4 | Correct |
| 62 | 1 | 5/3 | 1 to 3 | [4 to 8months] | Between 8 and 9 months | 6 | No | **No** | 1 to 2 | 1 | Correct |
| 63 | 1 | 5/3 | Four and over | ≥ 9 months | 9 months and more | 4 | No | **No** | 0 to 1 | 0 | Correct |
| 64 | 1 | 5/3 | 1 to 3 | [4 to 8months] | Between 8 and 9 months | 4 | No | **No** | 1 to 2 | No information | No |
| 65 | 1 | 9/3 | 1 to 3 | [4 to 8months] | Between 8 and 9 months | 5 | No | **No** | 1 to 2 | No information | No |
| 66 | 1 | 9/3 | 1 to 3 | [4 to 8months] | Between 8 and 9 months | 4 | No | **No** | 1 to 2 | No information | No |
| 67 | 1 | 9/3 | Four and over | [4 to 8months] | Between 8 and 9 months | 3 | No | **Yes** | 1 to 2 | 1 | Correct |
| 68 | 1 | 9/3 | 1 to 3 | [4 to 8months] | Between 8 and 9 months | 3 | No | **Yes** | 1 to 2 | 1 | Correct |
| 69 | 1 | 9/3 | 1 to 3 | ≥ 9 months | 9 months and more | 3 | No | **No** | 0 to 1 | 0 | Correct |
| 70 | 1 | 9/3 | 1 to 3 | ≥ 9 months | 9 months and more | 6 | No | Yes | 0 to 1 | 1 | Correct |
| 71 | 1 | 9/3 | 1 to 3 | [4 to 8months] | Between 8 and 9 months | 4 | No | **No** | 1 to 2 | 0 | No |
| 72 | 1 | 9/3 | Four and over | [4 to 8months] | Between 8 and 9 months | 3 | No | **Unknown** | 1 to 2 | No information | No |
| 73 | 1 | 9/3 | Four and over | [4 to 8months] | Between 4 and 6 months | 2 | No | **Unknown** | 3 to 5 | No information | No |
| 74 | 1 | 12/3 | 1 to 3 | [4 to 8months] | Between 7 and 8 months | 3 | No | **Unknown** | 1 to 3 | No information | No |
| 75 | 1 | 12/3 | 1 to 3 | [4 to 8months] | Between 8 and 9 months | 4 | No | **No** | 1 to 2 | 0 | No |
| 76 | 1 | 12/3 | Four and over | [4 to 8months] | Between 8 and 9 months | 7 | No | **No** | 1 to 2 | 0 | No |
| 77 | 1 | 12/3 | 1 to 3 | [4 to 8months] | Between 8 and 9 months | 3 | No | **No** | 1 to 2 | 0 | No |
| 78 | 1 | 12/3 | 1 to 3 | [4 to 8months] | Between 7 and 8 months | 4 | No | **Yes** | 1 to 3 | 2 | Correct |
| 79 | 1 | 12/3 | 1 to 3 | [4 to 8months] | Between 7 and 8 months | 3 | No | **Unknown** | 1 to 3 | No information | No |
| 80 | 1 | 12/3 | 1 to 3 | [4 to 8months] | Between 7 and 8 months | 4 | No | **Unknown** | 1 to 3 | No information | No |
| 81 | 1 | 12/3 | 1 to 3 | [4 to 8months] | Between 4 and 6 months | 3 | No | **Yes** | 3 to 5 | 4 | Correct |
| 82 | 1 | 12/3 | 1 to 3 | [4 to 8months] | Between 7 and 8 months | 2 | No | **Unknown** | 1 to 3 | No information | No |
| 83 | 1 | 12/3 | 1 to 3 | [4 to 8months] | Between 8 and 9 months | 7 | No | **No** | 1 to 2 | 0 | No |
| 84 | 1 | 12/3 | Four and over | [4 to 8months] | Between 7 and 8 months | 2 | No | **Unknown** | 1 to 3 | No information | No |
| 85 | 1 | 13/3 | Four and over | [4 to 8months] | Between 4 and 6 months | 3 | No | **Unknown** | 3 to 5 | No information | No |
| 86 | 1 | 13/3 | Four and over | [4 to 8months] | Between 8 and 9 months | 3 | No | **Yes** | 1 to 2 | 2 | Correct |
| 87 | 1 | 13/3 | 1 to 3 | ≥ 9 months | 9 months and more | 4 | No | **No** | 0 to 1 | 0 | Correct |
| 88 | 1 | 13/3 | Four and over | [4 to 8months] | Between 7 and 8 months | 7 | No | **Yes** | 1 to 3 | 2 | Correct |
| 89 | 1 | 13/3 | 1 to 3 | ≥ 9 months | 9 months and more | 5 | No | **No** | 0 to 1 | 0 | Correct |
| 90 | 1 | 13/3 | 1 to 3 | [4 to 8months] | Between 8 and 9 months | 3 | No | **No** | 1 to 2 | 0 | No |
| 91 | 1 | 13/3 | Four and over | ≥ 9 months | 9 months and more | 5 | No | **No** | 0 to 1 | 0 | Correct |
| 92 | 1 | 13/3 | 1 to 3 | [4 to 8months] | Between 8 and 9 months | 3 | No | **Unknown** | 1 to 2 | No information | No |
| 93 | 1 | 13/3 | 1 to 3 | ≥ 9 months | 9 months and more | 4 | No | **No** | 0 to 1 | 0 | Correct |
| 94 | 1 | 14/3 | 1 to 3 | [4 to 8months] | Between 8 and 9 months | 1 | No | **Yes** | 1 to 2 | 1 | Correct |
| 95 | 1 | 14/3 | 1 to 3 | [4 to 8months] | Between 4 and 6 months | 2 | No | **Unknown** | 3 to 5 | No information | No |
| 96 | 1 | 14/3 | Four and over | [4 to 8months] | Between 7 and 8 months | 4 | No | **Yes** | 1 to 3 | 2 | Correct |
| 97 | 1 | 14/3 | 1 to 3 | [4 to 8months] | Between 7 and 8 months | 3 | No | **Unknown** | 1 to 3 | No information | No |
| 98 | 1 | 14/3 | 1 to 3 | [4 to 8months] | Between 4 and 6 months | 3 | No | **Unknown** | 3 to 5 | No information | No |
| 99 | 1 | 14/3 | 1 to 3 | ≥ 9 months | 9 months and more | 5 | No | Yes | 0 to 1 | 1 | Correct |
| 100 | 1 | 14/3 | 1 to 3 | ≥ 9 months | 9 months and more | 3 | No | Yes | 0 to 1 | 1 | Correct |
| 101 | 2 | 23/3 | Four and over | [4 to 8months] | Between 7 and 8 months | 4 | Yes | Yes | 1 to 3 | 2 | Correct |
| 102 | 2 | 23/3 | Four and over | ≥ 9 months | 9 months and more | 3 | No | No | 0 to 1 | 0 | Correct |
| 103 | 2 | 23/3 | 1 to 3 | [4 to 8months] | Between 7 and 8 months | 5 | Yes | Yes | 1 to 3 | 2 | Correct |
| 104 | 2 | 23/3 | Four and over | ≥ 9 months | 9 months and more | 5 | No | No | 0 to 1 | 0 | Correct |
| 105 | 2 | 23/3 | Four and over | ≥ 9 months | 9 months and more | 5 | No | Yes | 0 to 1 | 1 | Correct |
| 106 | 2 | 23/3 | Four and over | ≥ 9 months | 9 months and more | 5 | No | No | 0 to 1 | 0 | Correct |
| 107 | 2 | 28/3 | Four and over | ≥ 9 months | 9 months and more | 3 | Yes | No | 0 to 1 | 0 | Correct |
| 108 | 2 | 28/3 | 1 to 3 | ≥ 9 months | 9 months and more | 4 | Yes | No | 0 to 1 | 0 | Correct |
| 109 | 2 | 28/3 | Four and over | ≥ 9 months | 9 months and more | 5 | Yes | No | 0 to 1 | 0 | Correct |
| 110 | 2 | 28/3 | Four and over | [4 to 8months] | Between 4 and 6 months | 4 | No | Yes | 3 to 5 | 4 | Correct |
| 111 | 2 | 28/3 | Four and over | [4 to 8months] | Between 7 and 8 months | 4 | Yes | Yes | 1 to 3 | 2 | Correct |
| 112 | 2 | 28/3 | 1 to 3 | ≥ 9 months | 9 months and more | 3 | Yes | Yes | 1 to 2 | 1 | Correct |
| 113 | 2 | 28/3 | Four and over | [4 to 8months] | Between 7 and 8 months | 1 | Yes | Yes | 1 to 3 | 2 | Correct |
| 114 | 2 | 28/3 | Four and over | [4 to 8months] | Between 8 and 9 months | 4 | No | Yes | 1 to 2 | 1 | Correct |
| 115 | 2 | 3/4 | Four and over | [4 to 8months] | Between 8 and 9 months | 5 | Yes | Yes | 1 to 2 | 1 | Correct |
| 116 | 2 | 3/4 | 1 to 3 | [4 to 8months] | Between 4 and 6 months | 1 | Yes | Yes | 3 to 5 | 3 | Correct |
| 117 | 2 | 3/4 | 1 to 3 | [4 to 8months] | Between 8 and 9 months | 5 | Yes | Yes | 1 to 2 | 1 | Correct |
| 118 | 2 | 3/4 | 1 to 3 | [4 to 8months] | Between 4 and 6 months | 3 | No | Yes | 3 to 5 | 5 | Correct |
| 119 | 2 | 3/4 | 1 to 3 | [4 to 8months] | Between 7 and 8 months | 3 | Yes | Yes | 1 to 3 | 2 | Correct |
| 120 | 2 | 3/4 | Four and over | ≥ 9 months | 9 months and more | 5 | Yes | No | 0 to 1 | 0 | Correct |
| 121 | 2 | 3/4 | Four and over | ≥ 9 months | 9 months and more | 5 | Yes | No | 0 to 1 | 0 | Correct |
| 122 | 2 | 4/4 | 1 to 3 | [4 to 8months] | Between 4 and 6 months | 2 | No | Yes | 3 to 5 | 4 | Correct |
| 123 | 2 | 4/4 | Four and over | [4 to 8months] | Between 4 and 6 months | 3 | Yes | Yes | 3 to 5 | 3 | Correct |
| 124 | 2 | 4/4 | Four and over | ≥ 9 months | 9 months and more | 5 | No | No | 0 to 1 | 0 | Correct |
| 125 | 2 | 4/4 | 1 to 3 | ≥ 9 months | 9 months and more | 6 | Yes | Yes | 0 to 1 | 1 | Correct |
| 126 | 2 | 4/4 | Four and over | [4 to 8months] | Between 4 and 6 months | 3 | Yes | Yes | 3 to 5 | 4 | Correct |
| 127 | 2 | 4/4 | 1 to 3 | ≥ 9 months | 9 months and more | 8 | Yes | No | 0 to 1 | 0 | Correct |
| 128 | 2 | 4/4 | 1 to 3 | [4 to 8months] | Between 4 and 6 months | 3 | Yes | Yes | 3 to 5 | 5 | Correct |
| 129 | 2 | 4/4 | 1 to 3 | ≥ 9 months | 9 months and more | 6 | Yes | No | 0 to 1 | 0 | Correct |
| 130 | 2 | 4/4 | Four and over | [4 to 8months] | Between 4 and 6 months | 3 | Yes | Yes | 3 to 5 | 3 | Correct |
| 131 | 2 | 4/4 | 1 to 3 | ≥ 9 months | 9 months and more | 2 | Yes | No | 0 to 1 | 0 | Correct |
| 132 | 2 | 6/4 | Four and over | [4 to 8months] | Between 7 and 8 months | 5 | No | Yes | 1 to 3 | 1 | No |
| 133 | 2 | 6/4 | 1 to 3 | [4 to 8months] | Between 4 and 6 months | 1 | Yes | Yes | 3 to 5 | 4 | Correct |
| 134 | 2 | 6/4 | Four and over | [4 to 8months] | Between 4 and 6 months | 5 | Yes | Yes | 3 to 5 | 3 | Correct |
| 135 | 2 | 6/4 | Four and over | [4 to 8months] | Between 4 and 6 months | 2 | Yes | Yes | 3 to 5 | 4 | Correct |
| 136 | 2 | 6/4 | 1 to 3 | [4 to 8months] | Between 4 and 6 months | 2 | Yes | Yes | 3 to 5 | 5 | Correct |
| 137 | 2 | 6/4 | 1 to 3 | [4 to 8months] | Between 7 and 8 months | 2 | No | Yes | 1 to 3 | 1 | No |
| 138 | 2 | 8/4 | Four and over | ≥ 9 months | 9 months and more | 4 | Yes | No | 0 to 1 | 0 | Correct |
| 139 | 2 | 8/4 | 1 to 3 | [4 to 8months] | Between 4 and 6 months | 2 | Yes | Yes | 3 to 5 | 4 | Correct |
| 140 | 2 | 8/4 | Four and over | [4 to 8months] | Between 7 and 8 months | 3 | No | Yes | 1 to 3 | 2 | Correct |
| 141 | 2 | 8/4 | 1 to 3 | [4 to 8months] | Between 7 and 8 months | 2 | Yes | Yes | 1 to 3 | 3 | Correct |
| 142 | 2 | 11/4 | 1 to 3 | [4 to 8months] | Between 4 and 6 months | 3 | Yes | Yes | 3 to 5 | 3 | Correct |
| 143 | 2 | 11/4 | Four and over | [4 to 8months] | Between 7 and 8 months | 6 | Yes | Yes | 1 to 3 | 1 | Correct |
| 144 | 2 | 11/4 | 1 to 3 | [4 to 8months] | Between 7 and 8 months | 3 | Yes | Yes | 1 to 3 | 2 | Correct |
| 145 | 2 | 11/4 | Four and over | ≥ 9 months | 9 months and more | 5 | No | No | 0 to 1 | 0 | Correct |
| 146 | 2 | 11/4 | 1 to 3 | [4 to 8months] | Between 4 and 6 months | 3 | No | Yes | 3 to 5 | 3 | Correct |
| 147 | 2 | 16/4 | 1 to 3 | [4 to 8months] | Between 4 and 6 months | 3 | Yes | Yes | 3 to 5 | 3 | Correct |
| 148 | 2 | 16/4 | 1 to 3 | [4 to 8months] | Between 4 and 6 months | 3 | Yes | Yes | 3 to 5 | 4 | Correct |
| 149 | 2 | 16/4 | Four and over | ≥ 9 months | 9 months and more | 4 | No | No | 0 to 1 | 0 | Correct |
| 150 | 2 | 16/4 | 1 to 3 | [4 to 8months] | Between 4 and 6 months | 3 | Yes | Yes | 3 to 5 | 5 | Correct |
| 151 | 2 | 16/4 | 1 to 3 | [4 to 8months] | Between 4 and 6 months | 3 | Yes | Yes | 3 to 5 | 4 | Correct |
| 152 | 2 | 16/4 | Four and over | [4 to 8months] | Between 4 and 6 months | 2 | Yes | Yes | 3 to 5 | 4 | Correct |
| 153 | 2 | 17/4 | 1 to 3 | ≥ 9 months | 9 months and more | 2 | No | No | 0 to 1 | 0 | Correct |
| 154 | 2 | 17/4 | 1 to 3 | [4 to 8months] | Between 7 and 8 months | 2 | No | Yes | 1 to 3 | 3 | Correct |
| 155 | 2 | 17/4 | 1 to 3 | [4 to 8months] | Between 4 and 6 months | 3 | No | Yes | 3 to 5 | 5 | Correct |
| 156 | 2 | 17/4 | 1 to 3 | ≥ 9 months | 9 months and more | 6 | Yes | No | 0 to 1 | 0 | Correct |
| 157 | 2 | 23/4 | 1 to 3 | [4 to 8months] | Between 7 and 8 months | 3 | Yes | Yes | 1 to 3 | 2 | Correct |
| 158 | 2 | 23/4 | 1 to 3 | [4 to 8months] | Between 7 and 8 months | 3 | Yes | Yes | 1 to 3 | 1 | Correct |
| 159 | 2 | 23/4 | 1 to 3 | ≥ 9 months | 9 months and more | 6 | Yes | No | 0 to 1 | 0 | Correct |
| 160 | 2 | 11/4 | 1 to 3 | [4 to 8months] | Between 7 and 8 months | 2 | No | Yes | 1 to 3 | 2 | Correct |
| 161 | 2 | 11/4 | 1 to 3 | ≥ 9 months | 9 months and more | 4 | No | Yes | 0 to 1 | 1 | Correct |
| 162 | 2 | 11/4 | 1 to 3 | [4 to 8months] | Between 4 and 6 months | 2 | Yes | Yes | 3 to 5 | 3 | Correct |
| 163 | 2 | 11/4 | 1 to 3 | [4 to 8months] | Between 7 and 8 months | 6 | Yes | Yes | 1 to 3 | 2 | Correct |
| 164 | 2 | 11/4 | 1 to 3 | [4 to 8months] | Between 7 and 8 months | 3 | Yes | Yes | 1 to 3 | 3 | Correct |
| 165 | 2 | 11/4 | 1 to 3 | ≥ 9 months | 9 months and more | 5 | Yes | Yes | 0 to 1 | 1 | Correct |
| 166 | 2 | 11/4 | 1 to 3 | [4 to 8months] | Between 4 and 6 months | 3 | Yes | Yes | 3 to 5 | 4 | Correct |
| 167 | 2 | 11/4 | 1 to 3 | ≥ 9 months | 9 months and more | 7 | No | No | 0 to 1 | 0 | Correct |
| 168 | 2 | 11/4 | 1 to 3 | [4 to 8months] | Between 4 and 6 months | 3 | No | Yes | 3 to 5 | 3 | Correct |
| 169 | 2 | 16/4 | 1 to 3 | [4 to 8months] | Between 4 and 6 months | 3 | Yes | Yes | 3 to 5 | 5 | Correct |
| 170 | 2 | 16/4 | 1 to 3 | [4 to 8months] | Between 4 and 6 months | 3 | No | Yes | 3 to 5 | 4 | Correct |
| 171 | 2 | 16/4 | 1 to 3 | [4 to 8months] | Between 4 and 6 months | 3 | No | Yes | 3 to 5 | 4 | Correct |
| 172 | 2 | 16/4 | Four and over | [4 to 8months] | Between 4 and 6 months | 1 | No | Yes | 3 to 5 | 5 | Correct |
| 173 | 2 | 16/4 | 1 to 3 | [4 to 8months] | Between 4 and 6 months | 3 | No | Yes | 3 to 5 | 3 | Correct |
| 174 | 2 | 16/4 | 1 to 3 | [4 to 8months] | Between 4 and 6 months | 3 | Yes | Yes | 3 to 5 | 5 | Correct |
| 175 | 2 | 16/4 | 1 to 3 | [4 to 8months] | Between 4 and 6 months | 2 | No | Yes | 3 to 5 | 3 | Correct |
| 176 | 2 | 16/4 | 1 to 3 | ≥ 9 months | 9 months and more | 4 | Yes | No | 0 to 1 | 0 | Correct |
| 177 | 2 | 16/4 | 1 to 3 | [4 to 8months] | Between 4 and 6 months | 3 | Yes | Yes | 3 to 5 | 3 | Correct |
| 178 | 2 | 16/4 | 1 to 3 | [4 to 8months] | Between 4 and 6 months | 3 | Yes | Yes | 3 to 5 | 3 | Correct |
| 179 | 2 | 16/4 | 1 to 3 | [4 to 8months] | Between 4 and 6 months | 2 | Yes | Yes | 3 to 5 | 3 | Correct |
| 180 | 2 | 17/4 | 1 to 3 | [4 to 8months] | Between 4 and 6 months | 1 | No | Yes | 3 to 5 | 5 | Correct |
| 181 | 2 | 17/4 | Four and over | ≥ 9 months | 9 months and more | 4 | No | No | 0 to 1 | 0 | Correct |
| 182 | 2 | 17/4 | 1 to 3 | [4 to 8months] | Between 4 and 6 months | 4 | No | Yes | 3 to 5 | 4 | Correct |
| 183 | 2 | 17/4 | Four and over | ≥ 9 months | 9 months and more | 2 | Yes | Yes | 0 to 1 | 1 | Correct |
| 184 | 2 | 17/4 | 1 to 3 | [4 to 8months] | Between 4 and 6 months | 3 | No | Yes | 3 to 5 | 4 | Correct |
| 185 | 2 | 17/4 | Four and over | [4 to 8months] | Between 7 and 8 months | 5 | No | Yes | 1 to 3 | 3 | Correct |
| 186 | 2 | 17/4 | 1 to 3 | [4 to 8months] | Between 7 and 8 months | 2 | Yes | Yes | 1 to 3 | 2 | Correct |
| 187 | 2 | 17/4 | 1 to 3 | [4 to 8months] | Between 7 and 8 months | 2 | No | Yes | 1 to 3 | 2 | Correct |
| 188 | 2 | 17/4 | 1 to 3 | [4 to 8months] | Between 4 and 6 months | 3 | Yes | Yes | 3 to 5 | 4 | Correct |
| 189 | 2 | 17/4 | 1 to 3 | ≥ 9 months | 9 months and more | 7 | No | No | 0 to 1 | 0 | Correct |
| 190 | 2 | 17/4 | 1 to 3 | ≥ 9 months | 9 months and more | 6 | Yes | Yes | 0 to 1 | 1 | Correct |
| 191 | 2 | 17/4 | 1 to 3 | [4 to 8months] | Between 4 and 6 months | 6 | No | Yes | 3 to 5 | 5 | Correct |
| 192 | 2 | 17/4 | 1 to 3 | [4 to 8months] | Between 4 and 6 months | 3 | No | Yes | 3 to 5 | 4 | Correct |
| 193 | 2 | 17/4 | Four and over | [4 to 8months] | Between 4 and 6 months | 2 | No | Yes | 3 to 5 | 3 | Correct |
| 194 | 2 | 23/4 | 1 to 3 | [4 to 8months] | Between 7 and 8 months | 3 | Yes | Yes | 1 to 3 | 2 | Correct |
| 195 | 2 | 23/4 | 1 to 3 | [4 to 8months] | Between 7 and 8 months | 5 | No | Yes | 1 to 3 | 2 | Correct |
| 196 | 2 | 23/4 | Four and over | ≥ 9 months | 9 months and more | 4 | No | Yes | 0 to 1 | 1 | Correct |
| 197 | 2 | 23/4 | 1 to 3 | ≥ 9 months | 9 months and more | 4 | No | No | 0 to 1 | 0 | Correct |
| 198 | 2 | 23/4 | 1 to 3 | [4 to 8months] | Between 7 and 8 months | 3 | Yes | Yes | 1 to 3 | 2 | Correct |
| 199 | 2 | 27/4 | 1 to 3 | [4 to 8months] | Between 4 and 6 months | 3 | No | Yes | 3 to 5 | 4 | Correct |
| 200 | 2 | 27/4 | 1 to 3 | ≥ 9 months | 9 months and more | 6 | Yes | Yes | 0 to 1 | 1 | Correct |

PI: Principal investigator

(*) Group 1 before using the checklist; Group 2 after using the checklist

(**) The unknown condition match with the woman who was not responding to the phone call or who changed the noticed phone number

($) The grey colour in the background notice no new SP uptake by the woman, due to effective childbirth

(Ʌ) The comparison is seen as positive if the woman took more SP than what was theoretically scheduled; Negative if the difference between the due SP intake and the effective IPTp-SP uptake was different by two or more intakes ; equal if there was just a one-time difference or no difference between the planned and the effective SP intakes.
