# Supplementary material for: The provider’s checklist to improve pregnant women coverage by intermittent preventive malaria treatment in Mali: a pilot implementation study
Source: Malar J. 2021 Oct 16;20:402. doi: 10.1186/s12936-021-03940-7 (PMC8520273; doi:10.1186/s12936-021-03940-7)
Supplement: Supplementary file 4 — Additional file 4. English version of full face-to-face, open-ended questionnaire for participating pregnant women. [file 12936_2021_3940_MOESM4_ESM.docx]

**Additional file-4** English version of full face-to-face, open-ended questionnaire for participating pregnant women

##
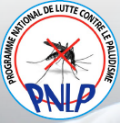


**QUESTIONNAIRE FOR PREGNANT WOMEN**

Woman surveyed N°___________/

Interview date: ________/________/ 201…

Group before checklist

Group after checklist

**SEEKING THE FREE AND INFORMED CONSENT OF PREGNANT WOMEN**

Hello,

My name's Issa DOUMBIA, student trainee at the National Malaria Control Programme (NMCP).

As part of the preparation of my thesis for specialization in Public Health and Hospital Management, we are conducting a study whose objective is to contribute to improving the knowledge of pregnant women on the use of intermittent preventive treatment for Sulfadoxine-Pyriméthamine (SP). To this end, we have prepared a quiz and we would like to ask you a few questions about your prenatal consultation with your attending physician today. There are no rights or wrong answers. We simply want to know your opinion. The answers you give us will be kept confidential.

Are you willing to participate in the study?

1 = Yes 2 = No

If No, stop interview

**Thank you for your answer!**

**Name of the gynecologist who performed the ANC**: ________________________

**Qualification of providers who have interacted with the pregnant woman**

1= General physician 2 = Midwife 3 = Other _______________

**I. Identification of the participant**

Initials (first and last names) participant: _____ / Hemoglobin b level:_____g/dl

**Marital status:**

1 = Married 3 = Divorced;

2 = Single 4 = Widowed.

**Age:** ………… year

**Interval of pregnancy age**

1 = Between 4 - 6 months 3 = More than 9 months

2 = Between 7 - 9 months 4 = Other (please specify)...........................

***Write the woman's other answers in the rectangle for each question***

**Question 1- What do you do for a job/profession?**

1 = Housewife 3 = Pupil/student

2 = Saleswoman 4 = Other (please specify)......

**Question 2 - Can we know your cultural background?**

1 = Bambara 4 = Dogon

2 = Bobo 5 = Senoufo

3 = Peulh 6= Other (specify)...................

**Question 3 - What is your level of education?**

1 = Illiterate 4 = Higher

2 = Primary 5 = Other .....................

3 = Secondary

**Question 4 - Obstetrical history (gesture: G).**

1= G1 4 = G4

2 = G2 5= G5

3 = G3 6 = G6 and more.

**Question 5 - During this pregnancy, how many ANCs have you had, including this visit?**

1 = ANC1 4 = ANC4

2 = ANC2 5= ANC5

3 = ANC3 6= ANC6 and more

**III - Knowledge of Malaria and Sulfadoxine-Pyriméthamine uptake**

**Question 6- Do you think you know about Malaria?**

***Write the woman's other answers in the rectangle for each question.***

1= Yes 2 = No

**If yes, can you give any symptoms of Malaria?**

1 = Fever

2 = Headache

3 = Nausea/vomiting

4 = General curves

5 = Other (specify) .................................................

**Question 7: How can it be prevented?**

***Write the woman's other answers in the rectangle for each question***

1= The use of LLINs

2 = The uptake of Sulfadoxine-Pyrimehamine

3 = The use of repellents

4 = Intra-household spraying

5 = Environmental health.

6 = Other (please specify)..............................................................

**Question 8 - Did the gynaecologist discuss this disease with you?**

1= Yes 2 = No

**Question 9- Have you received treatment for malaria? (Check on the prescription/carnet)**

1= Yes 2 = No

***Write the prescribed products in this rectangle***

***Madar/SP, iron or other***

**If Yes, what treatment did you receive?**

1 = Sulfadoxine-Pyriméthamine (SP)

2 = Quinine tablet

3 = CTA

4 = Serum and other antimalarial drugs

5 = Other (specify) ..................................

**If the drug given is Sulfadoxine-Pyrimethamine, have you taken the drug?**

1= Yes 2 = No

If No, why.............................................................................

**Question 10- How did you get it?**

1= Free of charge 2 = Buy

**Question 11- How many tablets did you take?**

1 = Three tablets

2 = Two tablets

3 = One tablet

4 = Three and more

**Question 12- Why does the pregnant woman have to take this medicine?**

1 = To protect the mother and her baby

2 = Protect the baby only

3 = Protect the mother only

4 = Other (specify) ........................................

**Question 13 - Was this the first time today that you have used this medicine?**

1= Yes 2 = No

**Question 14 - Based on what you were told during your visit, are there any special conditions to be observed when taking SP?**

1= Yes 2 = No

***Write the woman's other answers in the rectangle for each question***

If Yes, which ones?

1 = In the presence of the health worker

2 = Taken on an empty stomach or with food

3 = Do not know

4= No one explained anything to me

If No, to the best of your knowledge, how is SP taken?

**Question 15 - When do you have to start SP again?**

1 = Every month during ANC

2 = Two months after today's dose

3 = Three months later

4 = Daily

5 = Does not take anymore

**Question 16 - Can you list any side effects related to taking SP?**

1 = Vomiting

2 = Rash

3 = Jaundice

4 = Movement disorders

5 = Other (specify) .....................................

**Question 17 - What measures would you take in case of vomiting, rashes, jaundice, and movement disorders?**

1 = Go to the nearest health centre

2 = Stay home

3 = Does nothing

4 = Do not know

5- They did not explain anything about it to me

**Question 18 - Is it important that you understand everything the gynaecologist has told you?**

***Write the woman's other answers in the rectangle for each question***

1 = Very important

2 = More or less important

3 = Slightly important

4 = Not at all important

**Question 19 - Is it important that the gynaecologist gives you more time to talk about malaria prevention?**

1 = Very important

2 = More or less important

3 = Slightly important

4 = Not at all important

5 = Do not know.

**Question 20 - When do you have to come back for the next visit (in case of an appointment set by the provider, please confirm in the ANC booklet)?**

1 = In one month

2 = In two months

3 = In three months

4 = No appointment

**Question 21 - Why are you coming back?**

1 = Because I started ANC here

2 = Because the provider is competent

3 = Because the provider took good care of me

4 = Because I know someone here

5- Because free malaria medicine is available here

6- Because you can find the free mosquito net here

7 = Other (specify)....................

***Thanks a lot for your time!***
