# Supplementary material for: The provider’s checklist to improve pregnant women coverage by intermittent preventive malaria treatment in Mali: a pilot implementation study
Source: Malar J. 2021 Oct 16;20:402. doi: 10.1186/s12936-021-03940-7 (PMC8520273; doi:10.1186/s12936-021-03940-7)
Supplement: Supplementary file 5 — Additional file 5. Study method additional information. [file 12936_2021_3940_MOESM5_ESM.docx]

**Additional file-5** Study method additional information

**Description of the implementation process**

The reason for excluding 16 women from the participation of the study are:

**Excluded from the study in the First Group (n= 03)**

01 Pregnant women from outside Commune VI of the district hospital.

01 Pregnant women who visited the gynecologist in their 3rd month of pregnancy because of a precious pregnancy and who presented themselves to the investigator with a first ultrasound report and biology tests that had not been carried out.

01 Pregnant women who expressed an allergy to SP and cotrimoxazol.

**Excluded from the study is the Second Group (n= 04)**

02 Pregnant women from outside Commune VI of the district hospital.

01 pregnancy who visited the gynecologist in their 3rd month of pregnancy because.

01 Pregnant women who expressed an allergy to SP and Amoxicillin.

09 Pregnant women refused to participate due to family obligations but were not included in the analysis.

768 Pregnant women were not seen by the needed doctor to respect the selection ratio per doctor.

When the first 100 participants were recruited, none of the gynaecologists was aware of the existence of the checklist-provider as a reminder tool. The gynaecologists were asked to invite pregnant women to meet the interviewer and to decide whether they wanted to participate or not in a malaria study. The checklist was only presented to the gynaecologists during the second phase of the study. They were invited to recruit another 100 women under the same conditions, in order to neutralize any confounding factor linked to the communication style that could be different between the four gynaecologists providers and to be able to measure the effect of the checklist in pregnant women's knowledge of IPTp with SP with comparison to each gynaecologist groups.

Following the plan for organizing the activities of the gynaecology unit, each gynaecologist was assigned one day of consultations, including ANC for women with at-risk pregnancies. The study investigator monitored this schedule during the enrolment of women according to the eligibility criteria. The first 100 pregnant women were enrolled in the first phase with different proportions of each gynaecologist A, B, C and D. Then in the second phase, the same proportions were respected to enrol the second 100 women with a difference of the effect of the checklist on pregnant women's knowledge of IPT with SP.

The gynaecologists were asked to use the checklist to remind themselves of the ten standard messages to be given to pregnant women about malaria knowledge and the use of IPT with SP. They were instructed to tick the boxes on the checklist when the explanations were effectively given face-to-face (without necessarily having to follow the chronological order of the items mentioned). In the end, the visiting woman was invited to meet the study investigator to participate in the questionnaire just after leaving the gynaecologist's prenatal consultation. (going first to the drug unit and after receiving the noticed treatments, the community health worker showed the room localization of the study investigator).

With or without using the checklist, all gynaecologists pointed out to all pregnant women two prescriptions, one of which contained only the Sulfadoxine-Pyrimethamine (SP) to be received free of charge in the Drug unit dispensing room from the nurse. Moreover, the other prescription containing the supplementary drugs not available and that should be paid from any private pharmacy.

In the drug unit dispensing room, IPTp-SP should be taken with direct observation by the community health worker and was recorded in the register for follow-up. In practice, the nurse gives to the pregnant woman a dose of IPT-SP under direct observation. Three doses are taken at one time as early as possible during the second trimester (13^th^ to 16^th^ weeks) of the woman's pregnancy during each scheduled prenatal consultation until the time of delivery. That doses provided are administered at least 30 days after the last uptake for a safety issue. This treatment could be given on fasting or with food.

**NB:** It is after the administration of IPTp-SP uptake that the drug dispensing showed the room where the study investigator could enroll the woman for the study if she accepted to participate and signed the informed consent
